# An Electrochemical and Spectroscopic Study of Surfaces on Bronze Sculptures Exposed to Urban Environment

**DOI:** 10.3390/ma14082063

**Published:** 2021-04-20

**Authors:** Dajana Mikić, Helena Otmačić Ćurković, Tadeja Kosec, Neven Peko

**Affiliations:** 1 Research Laboratory for Corrosion Engineering and Surface Protection, Faculty of Chemical Engineering and Technology, University of Zagreb, HR 10000 Zagreb, Croatia; dmikic@fkit.hr; 2 Laboratory for Metals, Corrosion and Anticorrosion Protection, Slovenian National Building and Civil Engineering Institute, SI 1000 Ljubljana, Slovenia; tadeja.kosec@zag.si; 3 Conservation-Restoration Workshop, Sisak City Museum, HR 44000 Sisak, Croatia; neven.peko@gmail.com

**Keywords:** bronze, patina, spectroscopy, electrochemical behavior

## Abstract

Polluted urban environment enhances dissolution of patina and underlying bronze material of recent and historical bronze sculptures exposed outdoors. In this work, two bronze statues, situated in one of the most polluted Croatian cities, were examined in order to characterize composition of patina and its electrochemical stability. The composition of patina on several positions on each sculpture was determined by EDS, Raman spectroscopy, and FTIR measurements. Electrochemical impedance spectroscopy measurements were conducted in order to evaluate the corrosion stability of both patina and underlying bronze. Results obtained in this work show that the two examined bronze sculptures were covered with patina layer that was mainly composed of copper sulfides and sulphates, which is in accordance with the high concentrations of H_2_S and SO_2_ in the atmosphere. However, the variations in the appearance of FTIR and Raman spectra revealed that the amount of each species differed from spot to spot, as well as the fact that other compounds, such as carbonates, were present at some areas. This difference in patina composition was reflected in electrochemical behavior as observed by electrochemical impedance spectroscopy.

## 1. Introduction

Bronze has been used from ancient times for decorative and artistic objects. Even though bronze is known to be a relatively corrosion-resistant alloy, a significant decay, in terms of surface recession and loss of details in artefacts, has been observed on bronze objects exposed outdoors. Copper and its alloys spontaneously form a thin layer of brown-red oxide, cuprite Cu_2_O. In polluted aggressive atmospheres, the secondary corrosion products form that might change the color from dark brown to greenish tones due to the formation of various sulphate, chloride, or carbonate copper species [1,2,3]. Polluted urban environment enhances the dissolution of patina and underlying bronze material. The rate of patina dissolution depends on its composition and morphology as well as on the environment corrosivity.

Many studies have been conducted with the aim to understand the mechanism of corrosion of copper and its alloys in atmospheres containing various pollutants such as H_2_S, SO_2_, NO_2_, and O_3_ [4,5,6,7]. Laboratory studies in controlled conditions, with known pollutant concentration and wetting conditions provide important information on initial corrosion processes and patina formation. Various experimental setups have been arranged with an aim to simulate actual outdoor corrosion conditions. For example, a thorough study on dissolution of bronze and formation of corrosion products in conditions simulating exposure of sheltered (wet and dry method) and unsheltered (dropping method) parts of sculpture was conducted by Masi et al. [8]. It was found that such methods can produce a patina similar to the ones naturally formed with typical indices of decuprification of bronze due to selective dissolution of copper and accompanied to the formation of a tin-species network within the corroded structure. However, laboratory experiments usually last for several weeks [9], while patina on outdoor statues forms for many years. In that period mechanism of patina formation can change due to the changes in concentration of atmospheric pollutants or increase in patina thickness layer. Several studies have been conducted on copper and bronze coupons exposed to outdoor atmosphere for a couple of years with simultaneous monitoring of atmospheric conditions [2,10,11]. Such studies provide valuable information on gradual patina formation with respect to patina composition and thickness. Evolution of patina layer is also followed by the change of base metal corrosion rate in time. Still, even the longest studies on coupons lasted for maximum 16 years [10], while the real objects of cultural heritage remain exposed corrosive atmospheres for decades. For this reason, studies on copper and bronze objects with patina layer formed for many years are important for understanding corrosion processes on a long-term scale [12]. For example, Morcillo et al. [13] examined centuries-old patinated roof tile by various spectroscopic and microscopic investigations. Patina composition profile through the layer was related to the changes in corrosivity of the atmosphere due to the variation of SO_2_ concentration in time.

The research on patinated bronze objects is often focused on examination of patina layer alone. Various spectroscopic techniques are indispensable for characterization of patina composition; however, electrochemical techniques offer both possibility of patina composition and reactivity determination. For this purpose, various electrochemical techniques are employed, such as voltametric techniques [14,15,16] applied with an aim to determine the composition of patina and electrochemical impedance spectroscopy (EIS) measurements on patina powders inserted into the cavity microelectrode in order to determine patina reactivity [17,18]. Besides examining the patina alone, studies are also conducted on patinated bronze samples. In such research, EIS has become an indispensable, nondestructive technique for evaluation of corrosion properties of patinated surfaces. While such studies are easily conducted on bronze plates, use of an electrochemical cell with electrolyte can be challenging on curved and nonhorizontal areas of sculptures [19]. Solutions have been developed that involve the use of sponges or fabrics soaked with appropriate electrolyte [20]. Another possible replacement for the standard setup are gel electrolytes. Several studies on bare and coated bronzes have been conducted with gel based on agar [21,22,23,24] containing artificial rain solution. It has been shown that in such medium good electrochemical response is achieved. Moreover, hydrogels based on poly(vinyl alcohol) [25] have been used for that purpose.

In this work, two casted bronze statues, situated in a polluted urban environment, were examined in order to characterize their electrochemical behavior and composition of patina. Studies were conducted on bronze sculptures situated in the city of Sisak which was for many years one of the most polluted cities in continental Croatia due to the emissions from nearby oil refinery and steel production plant, which resulted in high H_2_S and SO_2_ concentrations in the air. From available data for the period between 1991 and 2004, the average annual SO_2_ concentrations in the city center were reaching values up to 30 µg/m^3^, while the concentrations of H_2_S were up to 2 µg/m^3^. The average annual concentrations measured near the oil refinery (4 km from city center) were up to 50 µg/m^3^ for SO_2_ and up to 8 µg/m^3^ for H_2_S [26]. For the period before 1991, data are not available, but it is reasonable to assume that H_2_S and SO_2_ concentrations were similar, or higher, as observed for many European cities [27]. In recent years, decrease in H_2_S and SO_2_ concentration has been reported.

The aim of this work was to correlate the composition of patina with the corrosion resistance of the bronze at the particular position on the sculpture.

## 2. Materials and Methods

The sculptures examined in this work were exposed to polluted outdoor environment for more than 40 years and with no restoration works in last 30 years. The first sculpture, Nedovršena igra (NI—translation Unfinished game) by Gabriela Kolar, was exposed in 1965. It is placed in a park in Sisak city center. The position of the sculpture is inside a fountain. For several decades, the sculpture was exposed to sprinkling water, except for winter months, when the fountain was not in operation. However, the fountain stopped working several months before the measurements were conducted. The second sculpture, authored by Milena Lah, was exposed in 1974 at Memorial Place of Child Cemetery (MC) situated in the Sisak suburb. Both sculptures were casted in Ljevaonica umjetnina ALU foundry in Zagreb. MC is positioned about 3 km north from an oil refinery plant and a steel production plant (closed few years ago). The distance of NI from these plants is about 6 km.

**Figure 1:**
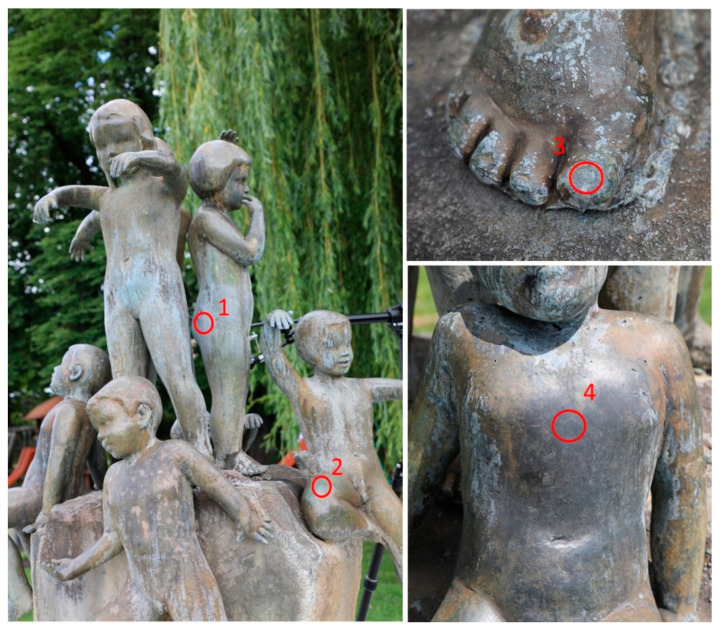
Sculpture *Nedovršena igra with indicated test areas 1–4*.

On each sculpture, several areas were selected for analysis. Selection was based on variation in surface appearance as well as on the position on the sculpture. Additionally, it was necessary to find sufficiently homogeneous and flat surface to conduct the planned research. On Nedovršena igra, 4 areas were chosen (Figure 1): area 1 (NI1)—vertical and sheltered area covered by bluish corrosion products; area 2 (NI2)—horizontal unsheltered area with compact green patina layer; area 3 (NI3)—horizontal area at the lower part of the statue, partially sheltered with bluish patina deposits; and area 4 (NI4)—vertical unsheltered area with dark grey patina layer.

**Figure 2:**
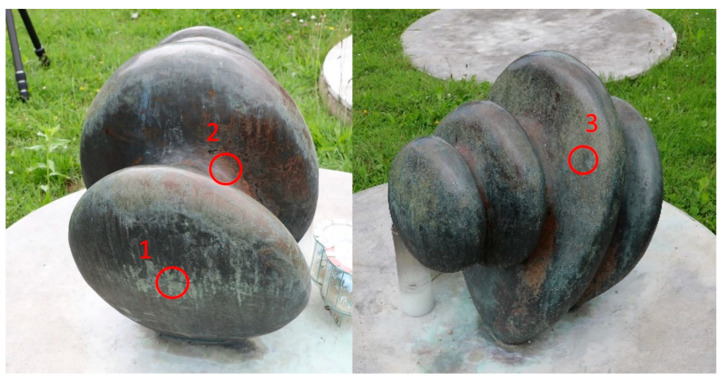
Sculpture at Memorial Cemetery with indicatedtest areas 1–3.

On the second examined sculpture (MC), 3 areas were selected for examination (Figure 2): area 1 (MC1)—vertical sheltered dark grey area covered by greenish patina; area 2 (MC2)—horizontal unsheltered red area; and area 3 (MC3)—vertical unsheltered dark grey area covered by greenish patina.

Measurements were conducted in a way wherein first EIS measurements were conducted, followed by X-ray fluorescence (XRF) and optical microscopy (OM), followed by scraping of patina from the surface for further analysis, including EDS, FTIR, and Raman spectroscopy.

Electrochemical impedance spectroscopy (EIS) measurements were conducted on several areas on each sculpture by using electrochemical cell with agar-based gel. Agar gelled electrolyte was prepared from 2.5% agar solution in simulated rainwater (0.2 g/L NaNO_3_, 0.2 g/L Na_2_SO_4_, and 0.2 g/L NaHCO_3_ adjusted to pH 6.5 with 5% H_2_SO_4_). Measurements were conducted using a three-electrode electrochemical cell with stainless steel wires as a pseudoreference and counter electrodes. Setup was adapted from the literature [21]. First, open circuit potential (OCP) was monitored for at least 20 min in order to ascertain that stationary conditions were achieved. EIS measurements were then conducted using Palm Sens4 potentiostat at OCP in the frequency range 100 kHz to 10 mHz and with 10 mV amplitude with 6 points per decade. Measurements were conducted on 5.31 cm^2^ surface area. One EIS measurement was acquired for each selected area. It is assumed that the examined surface area was sufficiently large enough to assure acquisition of representative and good quality EIS spectrum.

For fitting experimental EIS data to selected equivalent electrical circuits, ZSimpWin software was used.

Composition of patinated bronze was determined by X-ray fluorescence by using portable Olympus X-Ray Analyzer. Optical microscopy was conducted on selected spots by using Dino-Lite AM-7013MZT optical microscope.

The Raman spectra of patina powders were recorded by Horiba Yvon LabRAM HR spectrometer (France, 2009). The samples were irradiated with a green laser at λ = 514 nm. The scanning range was 50–4000 cm^−1^, each acquisition with an accumulation time of 20–35 s, depending on the number of counts. The background was not subtracted.

FTIR measurements were carried out by attenuated total reflectance Fourier transform infrared spectroscopy (ATR-FTIR) using a Spectrum One FTIR spectrometer from Perkin Elmer, with the scan range from 4000 to 650 cm^−1^, having a resolution of 0.5 cm^−1^. The results shown in this paper were averages of 25 scans. EDS analysis was performed with VEGA 3 SEM TESCAN equipped with X-ray energy-dispersive spectroscopy SEM imaging at an acceleration voltage of 10 kV.

## 3. Results and Discussion

### 3.1. Sculpture Nedovršena igra (Author Gabrijela Kolar—1965)

**Figure 3:**
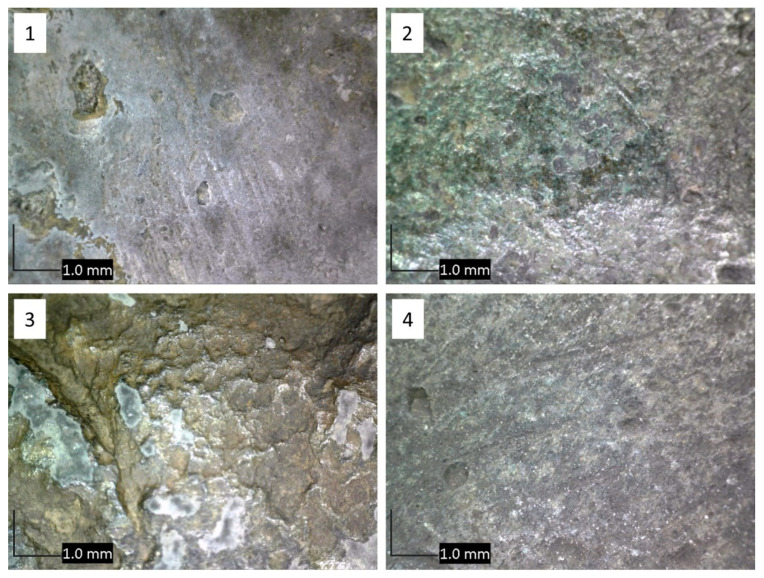
Optical microscopy of studied areas 1–4 on the NI sculpture, as shown in Figure 1.

The sculpture Nedovršena igra (NI) is made of quaternary Cu–Sn–Zn–Pb bronze. Optical microscopy revealed that the color and the surface morphology of patina at each area was quite different (Figure 3). XRF measurements were conducted at all areas of interest and the obtained compositions are shown in Table 1. Studies conducted by Robotti et al. [28] and Šatović et al. [29] showed that composition determined by XRF measurements on corroded bronze samples are influenced by the composition and thickness of corrosion products layer. For very thick patina layers, XRF results mainly show the composition of patina rather than composition of underlying bronze substrate. In this work, thickness of the patina layer was only qualitatively assessed during the patina scraping. The thickest patina layer was observed on NI1, followed by NI3, while the patina at NI2 and NI4 was much thinner.

Analyzed areas on bronze surface revealed the presence of Sn, Pb, and Zn, as well as some Fe. At several areas, significant amount of Al was also found. It is not uncommon that small amounts of Al are added during bronze casting [30], but such high Al content may also be a result of alumina deposition on bronze [10]. For many years, a steel production plant was operating in Sisak; such plants typically generate fly ashes that can contain up to 40% of alumina. In favor of this assumption is the fact that at point 1, which is a vertical surface, slightly sheltered from the wind, no Al was detected, while the highest amount of Al was observed at point 3, which is horizontal. At this rough surface, the deposition of airborne particles is more likely to have occurred. On the other hand, lack of Al at NI1, where patina layer appeared to be the thickest and XRF results were more influenced by patina composition than on other spots, may indicate that Al is present in a bulk alloy.

EDS examination of patina collected at point 1 of NI was performed in several spots and in principle two different compositions were observed. One with 53 wt % of Cu, 24 wt % Sn, 11 wt % Pb, 9 wt % of C, 3 wt % of S, and almost no oxygen content, and the other with a high amount of oxygen (29 wt %) and carbon (5 wt %), and a small amount of Sn (1.3 wt %), Ca (4.7 wt %), and Si (1.4 wt %). Aluminum was also present in some spots (max. 2 wt %), although it was not observed with XRF.

**Figure 4:**
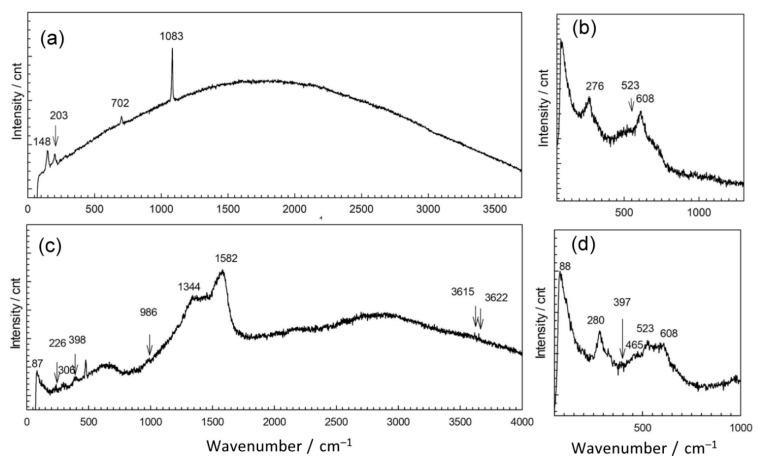
Raman spectra collected on patina samples from NI: point 1 (**a**), point 2 (**b**), point 3 (**c**), and point 4 (**d**).

In order to explain the observed differences in patina appearance, we sampled a small amount of patina powder and characterized it by Raman and ATR-FTIR spectroscopy. Raman spectra of corrosion products collected at point 1 showed the presence of several bands positioned at lower wavenumbers 148, 203, 702, and 1083 cm^−1^ (Figure 4 and Table 2). Similar spectra were recorded for different carbonate minerals [31]. For copper carbonate minerals, Raman spectrum exhibits bands at 144, 215, 739, and 1096 cm^−1^ (azurite) and 142, 205, 717, and 1096 cm^−1^ (malachite) [32,33]. Thus, Raman spectrum obtained for NI1 patina indicates that mixture of carbonate compounds was probably present. Taking into account the complex composition obtained by EDS, we found it was also possible that patina contained carbonates not only of copper but of other elements too.

**Figure 5:**
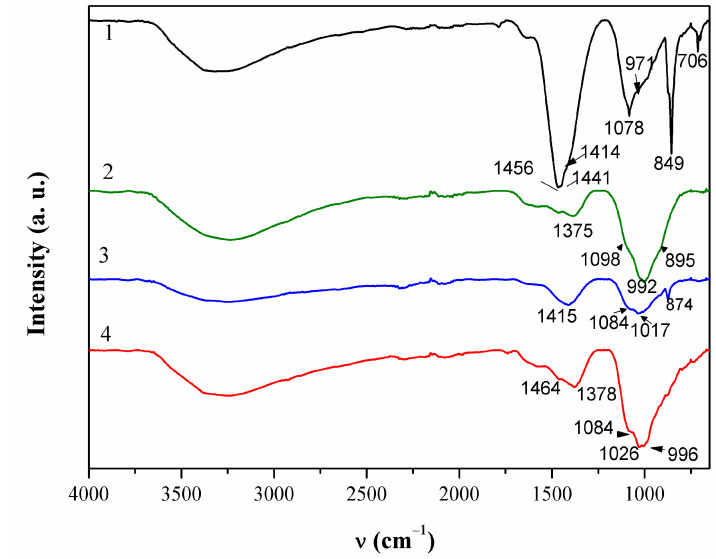
ATR-FTIR spectra collected on patina samples from NI.

ATR-FTIR spectrum of this patina sample is given in Figure 5. Broad bend in 3200–3500 cm^−1^ region can be attributed to OH stretching. Two bands were observed at 1456 and 1415 cm^−1^ that were probably related to the presence of carbonates [34].

Spectroscopic analysis showed that carbonates were present in the analyzed patina, while from EDS analysis, the strong enrichment in Sn was detected, which can point at the possible selective dissolution of copper commonly observed in corrosion of bronze at non-sheltered areas [35].

**Table 2:** Raman bands (cm^−1^), identified on patinas, analyzed at different positions of the statue NI (Figure 4) and literature data [32,36,37,38,39]. Letters next to a number denote strength of the band: vw (very weak), w (weak), s (strong), and vs (very strong).

NI1	NI2	NI3	NI4	Cu_2_O [8,37,38]	Cu_2_S [37,38]	Malachite [32,37]	Azurite [32]	Brochantite [36]	Atacamite [39]
		87	88 (s)						
148	144			153		142	144	141	
203						205	215		194
		226		220					236
	276 (s)		280 (s)		281		281		271
		306		309					
		398 (w)	397 (w)			398			363
	410 (w)						414	415	422
			465 (w)		472			467	
	523		523 (s)	523		531	540	517	
	608 (s)		608 (s)	628	603			608	
702						717			
		986						990	976
1083 (s)						1096	1095		
		1344 (s)				1364	1415		
		1582 (s)				1576			
		3615 (vw)							
		3622 (vw)							

The second examined position on NI had a well-adhered green patina layer (Figure 4b). EDS analysis showed some variations in patina composition; in some spots, an increase of Sn contents was observed, while the oxygen content was between 57 atom % and 61 atom %. Spots with lower Sn content had higher carbon and sulfur content. Raman spectra (Figure 4b) revealed bands at 144 cm^−1^ related to O–Cu–O bending, and at 523 and 608 cm^−1^, characteristic of M–O stretching vibrations, to Cu but also to Sn oxides [8]. Strong band positioned at 276 cm^−1^ points at possible presence of Cu_2_S, which also exhibits bands at 603 cm^−1^. The presence of sulphate compounds cannot be excluded as for brochantite and antlerite Raman spectrum exhibits high intensity band at 415 cm^−1^, as well as the bands at 141 and 608 cm^−1^ [36].

FTIR spectra (Figure 5) revealed a broad peak at 992 cm^−1^ that can be assigned to sulphate compounds, as well as the peak at 1098 cm^−1^ [40]. The peak at 1375 cm^−1^ indicated that some organic compounds may have been present too.

EDS analysis of the bluish patina from spot 3 on NI showed (Figure 1, spot 3) the presence of significant amounts of O and S (23 atom % of Cu, 58 atom % of O, and 16 atom % of S) in some spots, while in other spots high C content instead of S was observed. This could be either due to the presence of organic contaminants or carbonates in patina. Additionally, various amounts of Al (up to 4 atom %), Si, and Ca (up to 3 atom %) were observed.

Raman spectrum exhibited bands at 226, 380, and 479, and hindered band at 610 cm^−1^ as well as characteristic band at 978 cm^−1^ are similar to those observed in bronchantite and langite [36]. The bands above 3600 cm^−1^ are not commonly observed in Raman spectra of patina samples but have been found in complex silicate minerals such as chrysocolla [41]. Additional bands at 1334 and 1582 cm^−1^ could be due to the presence of graphite ashes. These bands could also originate from carbonates, which are likely to be present as 1415 cm^−1^ band was observed in FTIR spectrum. Still, taking in account that fountain water contained a small amount of chloride compounds, the presence of chloride patina cannot be excluded, since the bands at 226, 398, and 986 cm^−1^ could also have originated from chloride compounds such as atacamite.

Patina sampled in point 4 of NI also exhibited complex composition. Part of the powder contained up to 59 atom % of Sn and 38 atom % of O, while the other part was mainly composed of Cu (26 atom %), O (54 atom %), C (12 atom %), and S (3 atom %). Al was observed at all spots, but higher amounts were measured in spots containing mainly Cu and O, which would correspond to outer patina layer where weight percentage ratio was Cu/Al = 29:5.9. Such high Al content, compared to that observed by XRF, can be considered as a confirmation that Al was deposited from airborne particles, besides being present in a bulk alloy. EDS analysis of patina sampled from NI did not reveal Fe, and thus Fe content observed by XRF was related to its presence in alloy. Raman spectra (Figure 4b) recorded on several samples from the same point also showed some differences, but observed bands at 465, 523 and 608 cm^−1^ can be ascribed to presence of copper and tin oxides, whereas strong band at 280 cm^−1^ points to the presence of Cu_2_S [37,41]. On the other hand, FTIR spectrum (Figure 4) was similar to that obtained in point 2, which was ascribed to copper carbonates and sulphates.

Analysis of the patina samples from NI showed that natural patina form on the sculpture was very complex and contained various compounds that formed during 55 years of exposure to polluted atmosphere and splashing with fountain water. Moreover, each examined area exhibited different color and morphology due to the difference in composition. Patina in point 1 was more typical of patina found on outdoor bronzes in sheltered areas, with inner patina layer rich in Sn compounds and outer layer rich in copper corrosion products, mainly carbonates forming in contact with fountain water-leaking upper parts of the sculpture. On the other hand, points 2 and 4 represent unsheltered areas that come in contact with rainwater as well as fountain water. In these spots, enrichments of patina with tin oxides was observed. In point 3, bluish, easily removable patina was studied, being composed of copper sulfates and carbonates as well as silicates. Such patina is probably the result of dissolution of bronze in the upper parts of the sculpture and precipitation of corrosion products on the bottom of the statue.

### 3.2. Sculpture at Memorial Child Cemetery (MC) (Author Milena Lah—1974)

The second studied sculpture is made of quaternary Cu–Sn–Zn–Pb bronze (Table 3) with similar Sn content, lower Pb, and higher Fe content when compared to the first bronze sculpture. The presence of Fe usually means that bronze was casted from recycled alloys [30]. As in the case of Al, it was not clear if Fe content was only due to the initial content in alloy or if it was influenced by the pollution from the nearby steel factory. Studies were conducted at three points, as shown in Figure 2.

**Figure 6:**
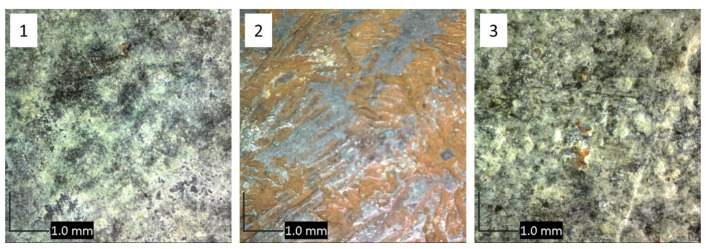
Optical microscopy of studied areas 1–3 on the MC sculpture, as shown in Figure 2.

Optical micrographs (Figure 6) showed relatively similar appearance of bronze surface in points 1 and 3 with outer layer of green patina and inner dark patina, while the surface in point 2 was covered by red and blue patina layers as observed by the naked eye and optical microscopy.

**Figure 7:**
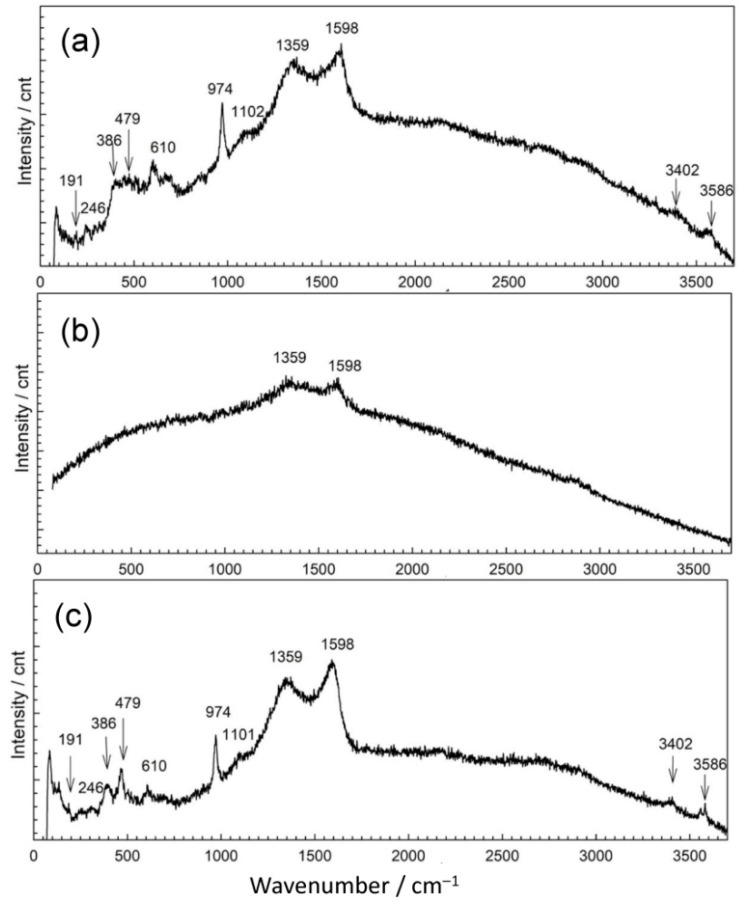
Raman spectra collected on patina samples from MC: position 1 (**a**), position 2 (**b**), and position 3 (**c**).

**Figure 8:**
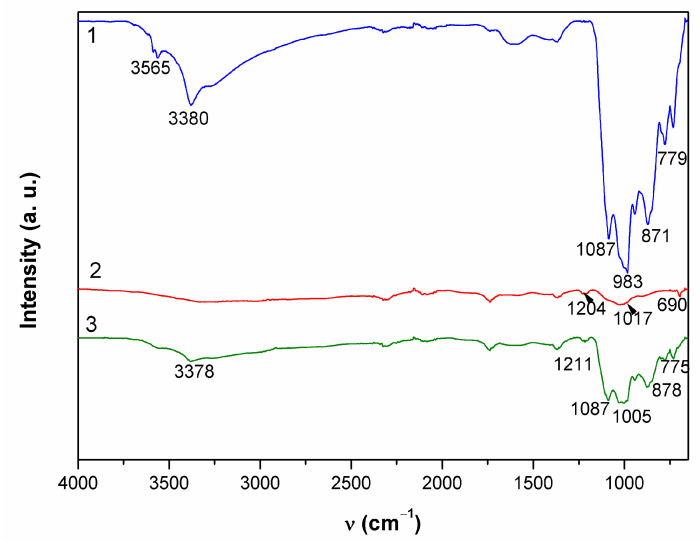
ATR-FTIR spectra collected on patina samples from MC.

In point 1, EDS revealed two distinctive patina compositions, one mainly containing copper (27 atom %), oxygen (59 atom %), sulfur (3 atom %), and carbon (10 atom %) with some remaining Al and Si. The second kind of patina composition observed had low oxygen content but high Sn content and some sulfur. In Raman spectrum shown in Figure 7a, bands at 191 cm^−1^; 246 cm^−1^ triplet band at 386, 479, and 610 cm^−1^; strong band at 974 cm^−1^; and broad band at 1102 cm^−1^, accompanied by characteristic bands at 3402 and 3586 cm^−1^, point to the presence of posnjakite or langite (Table 4). FTIR spectrum (Figure 8) also exhibited absorption maxima typical of copper sulphates [42,43].

The appearance of a second examined spot was much more different. It was placed on horizontal part of the sculpture and one must take in account possibility of mechanical removal of the loosely adhering patina from the surface if someone stepped on the sculpture. As observed in studies simulating bronze outdoor corrosion [8], Cu- and Zn-selective dissolution accompanied to the formation of a tin-species network within the corroded structure occur on quaternary bronzes and may result in formation of structures similar to those in Figure 6(2). Raman spectrum (Figure 7b) exhibited very few peaks, mainly related to M–O stretching vibrations. From the FTIR spectrum (Figure 8), we see that existence of some sulphate patina seems likely.

Both Raman (Figure 7c) and FTIR spectra (Figure 8) on patina from point 3 were similar to those from point 1, which leads to the conclusion of almost identical patina composition with inner layer mainly composed of sulfide compounds and with increased tin content, as well as outer layer with sulphate patina.

Cuprous sulfide found on analyzed patina could be due to initial artistic patination with liver of sulfur (K_2_S) or due to the presence of H_2_S in the atmosphere. The source of Cu_2_S presence could not be revealed. EDS analysis was conducted at several spots of each sampled patina powder, and various amounts of Fe and Al were observed for the same position. Sometimes there was Cu/Al or Cu/Fe ratio exceeding that observed by XRF, which would be in favor of their airborne deposition, and sometimes there was no Al and Fe, which would be in favor of its main source from the bulk alloy. Thus, it may be assumed that Al and Fe, observed by XRF, originated from both airborne particle deposition and the alloy itself.

### 3.3. Electrochemical Impedance Spectroscopy Studies

**Figure 9:**
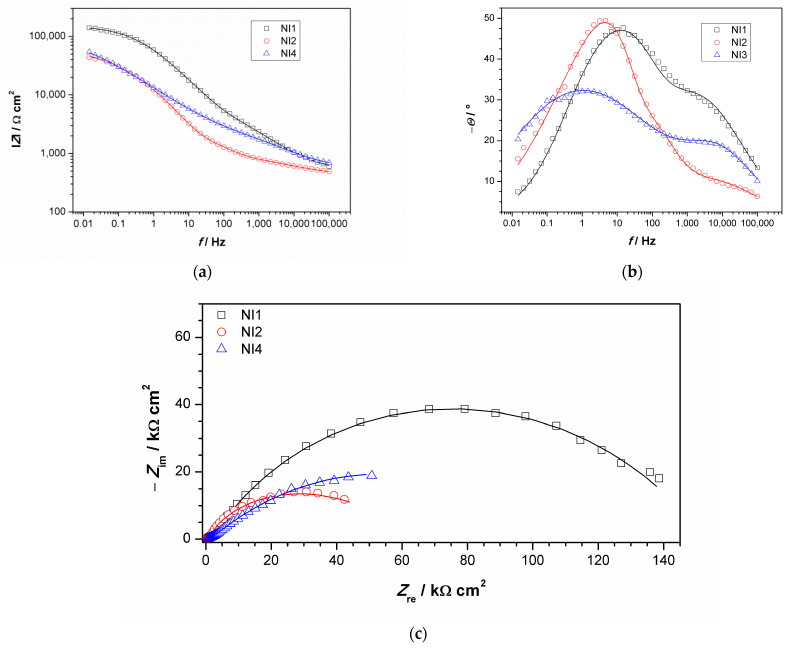
Impedance spectra obtained on NI: (**a**) impedance modulus Bode plot, (**b**) phase angle Bode plot, and (**c**) Nyquist plot. Symbols represent measured and lines fitted data.

EIS measurements were conducted using an agar cell. NI studies were conducted in points 1, 2 and 4, while point 3 did not have a sufficiently flat area to achieve good contact between the agar and sculpture surface. Obtained impedance spectra are given in Figure 9. Impedance modulus values at lowest measured frequency (usually 10 mHz) is often examined as a measure of material corrosion resistance, especially in the case of complicated EIS spectra obtained on patinated samples that are not easy to model with equivalent electrical circuits models. Survey of EIS measurements on copper and bronze patinas, conducted using contact probe setup [44], revealed that in the studied set of bronze objects, surfaces exposed to marine environment or those in rain-washed areas with bronchantite exhibit impedance modulus lower than 17 kΩ cm^2^, while the surfaces with the highest corrosion resistance exhibited impedance modulus between 0.3 and 1 MΩ·cm^2^. The composition of patina on these surfaces was similar to those with lower corrosion resistance. Higher impedance values were observed only on surfaces with remains of protective coating. Ramirez et al. [45] used agar-based electrolyte for studies of bronze sphinxes, wherein they examined two surface areas, one with dark patina with impedance moduls around 10–20 kΩ·cm^2^ and another with green patina layer exhibiting impedance modulus around 30–50 kΩ·cm^2^. In our study, spectrum obtained for NI1 exhibited the highest impedance modulus (0.14 M Ω·cm^2^) compared to other two surface areas. Indeed, such value of impedance modulus puts the examined surface in a group of patinated surfaces with higher corrosion resistance, i.e., the analyzed surface, with outer carbonate layer and inner tin enriched layer, which provides protection to underlying bronze substrate.

**Figure 10:**
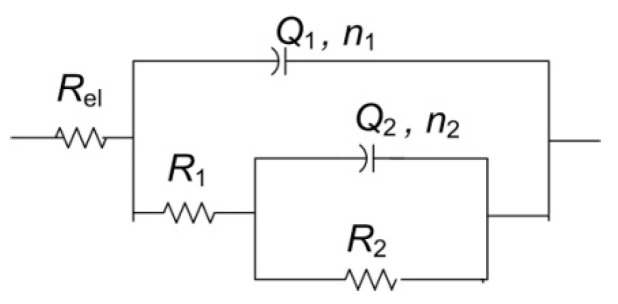
Electrical equivalent circuit used for analysis of EIS data in Figure 9.

For the other two examined spots, lower impedance modulus values were observed. All spectra exhibited two-phase angle maxima in Bode plot. Various EEC have been applied in the literature to describe EIS spectra obtained on patinated metals [19]. For spectra with two-phase angle maxima, usually a model with nested (R-Q) circuit is used, such as the circuit shown in Figure 10. In this model, R_el_ represents electrolyte resistance between working and reference electrode; high frequency data are described by R_1_, which represents corrosion product layer resistance, and Q_1_, which is constant phase element describing capacitive property of corrosion products layer; and the medium frequency part of the EIS spectra is represented by R_2_—charge transfer resistance, and Q_2_—constant phase element, describing double-layer capacitance, where n_1_ and n_2_ are coefficients describing the non-ideal capacitive behavior. Such a model is applicable for patina layers with pores through which an electrolyte can reach the metal surface. A similar model has been applied in other studies examining copper samples upon 3 years of exposure to atmospheric corrosion [46]. Sometimes the second R-Q couple is considered to be the response of the inner patina layer, rather than the response of bare bronze [47]. Considering the fact that EDS data point towards the existence of an inner tin oxide layer, such interpretation cannot be completely excluded, although low n_2_ values for NI1 and NI3 are in favor of the first interpretation.

Obtained impedance parameters are given in Table 5. Patina layer resistance is the highest for the NI1 spot, in sheltered area where composition is dominated by the presence of carbonates, while in unsheltered areas with dominantly sulphate (NI2) or sulfide patina, lower *R*_1_ value was observed. The charge transfer resistance value was also the highest for NI1 with the lowest *Q*_2_ value, which confirmed that this type of patina provided the best protection to underlying substrate. However, the optical microscopy of NI1 showed that the surface at this spot was not homogenous and that some defects in the structure existed. It should be taken in account that measurements were conducted with agar gel, and in a case of deeper craters in the surface, the wetting of the bottom of the craters might be limited. Thus, the observed resistance values were more representative of the area with compact patina layer. At two other examined spots, such defects were not observed.

**Figure 11:**
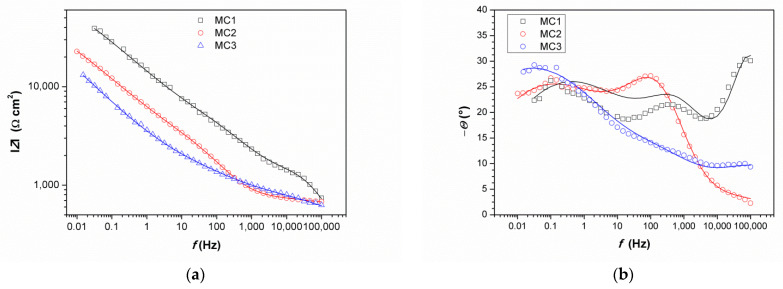

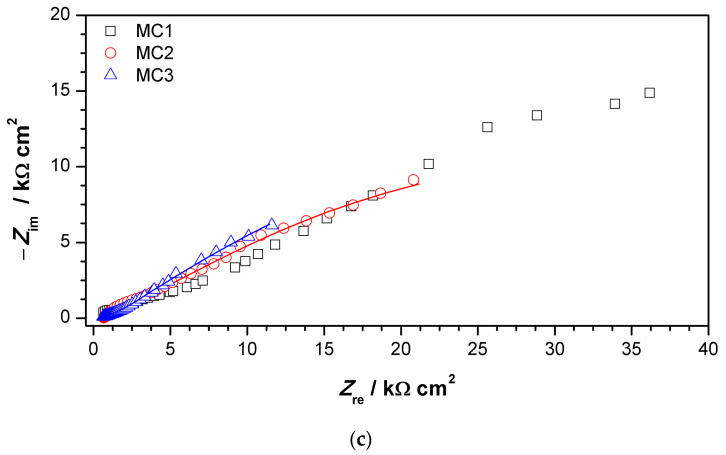
Impedance spectra obtained on MC: (**a**) impedance modulus Bode plot, (**b**) phase angle Bode plot, and (**c**) Nyquist plot. Symbols represent measured and lines fitted data.

**Figure 12:**
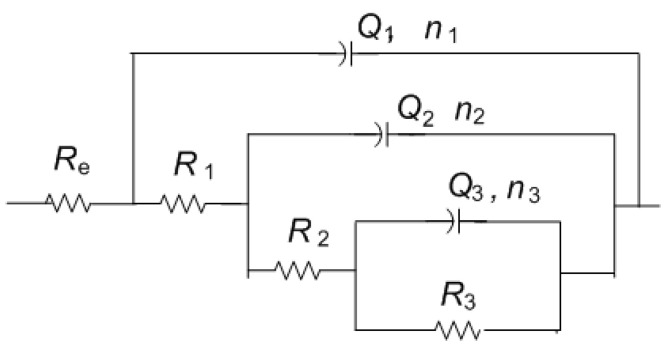
Electrical equivalent circuit used for analysis of EIS data in Figure 9.

Impedance spectra obtained on MC are given in Figure 11. It can be immediately observed that the values of impedance modulus at the lowest frequencies at all examined spots were lower than on NI. Additionally, these EIS spectra exhibited three phase angle maxima in Bode diagrams, more or less clearly resolved. Spectra were modeled with electrical equivalent circuit shown in Figure 12. It has additional time constant compared to the one in Figure 9 (R_3_-Q_3_). In our previous studies on chemical and electrochemical artificial patina, these two elements were allocated to the Faradaic resistance and Faradaic capacitance, implying the oxidation–reduction processes of the corrosion products (i.e., the outer patina layer) [48,49]. As can be seen from the data given in Table 4, n_3_ values obtained were quite low, around 0.5, indicating that this time constant was more likely related to diffusion processes inside the patina layer.

EIS parameters given in Table 6 showed that the resistance of patina layer on all studied spots at MC was lower compared to that on NI, as well as the charge transfer resistance. Patina layer resistance was the lowest for MC2 with the highest Q_1_ value, indicating the thinnest patina layer. The impedance at all MC spots was dominated by diffusion impedance, indicating the presence of narrow pores in the layer. For MC1, EIS parameters were not provided as it was not possible to obtain good fit between experimental data and model in lower frequency range.

The EIS results revealed that electrochemical behavior of patinas observed on NI and MC were different, which can be attributed to the difference in patina morphology and composition. Obtained impedance parameters pointed towards the presence of fine pores that limited the diffusion of O_2_ in MC patina layers, while the structure of patina on NI would be more compact but with the presence of bigger pores. Part of the explanation on such differences is surely related to the influence of fountain water on NI, as calcite and other carbonate minerals may induce plugging of fine pores. It is also interesting to notice that MC is closer to the oil refinery than NI, which is in the city center. Thus, MC was exposed to higher H_2_S concentrations, which may have also been the cause of lower corrosion resistance of MC. From the obtained results, it appears that areas with dominant carbonate patina exhibited higher corrosion resistance than those with sulfate or sulfide patina.

It is important to notice that similar chemical composition of patina at two different spots does not necessarily result in the same electrochemical response, as was observed for MC1 and MC3. Raman and FTIR spectra of patina sampled at these two points were almost identical, but EIS spectra were quite different. For MC1, impedance modulus values at all frequencies were several times higher than that of the MC3, and thus the corrosion resistance of the surface at point MC1 was higher. Differences were also observed in a shape of the phase angle plot. The possible reason for such difference could be patina layer thickness, as point 3 lies in an unsheltered area where dissolution of patina is more intense than on sheltered MC1 area where thicker patina layer may build. Increased corrosion resistance of bronze is often associated with the formation of the tin oxide layer. However, in our study, at all studied points, tin oxides were found by EDS, while XRF measurements did not reveal significant difference in Sn content between examined spots.

## 4. Conclusions

Spectroscopic and electrochemical studies were conducted on two bronze sculptures exposed to heavily polluted urban atmosphere. The patina and bronze surface were analyzed by XRF, EDS, Raman, and FTIR analysis. It was found that morphology and appearance of patina at different analyzed areas varied with the position at the bronze sculpture. Patina consisted of Cu and Sn oxides, and cuprous sulfide was found as well as copper sulphates, which were expected due to the presence of H_2_S, SO_2_, and SO_3_ in the atmosphere in the past years. However, the variations in the appearance of FTIR and Raman spectra revealed that the amount of each of these species differed from spot to spot, as well as the fact that that other compounds such as carbonates were present in some areas. This difference in patina composition influenced electrochemical behavior as observed by electrochemical impedance spectroscopy. Besides the composition alone, thickness and the morphology of the patina played an important role in corrosion resistance of the patinated bronze surfaces.

## Figures and Tables

**Table 1:** Composition of studied locations on NI sculpture (Figure 1) obtained by portable XRF.

Area/Composition wt %	1	2	3	4
Cu	71.7	69.0	65.9	68.5
Pb	9.9	8.7	7.1	9.6
Sn	8.9	10.0	10.4	8.1
Zn	7.4	3.9	4.4	6.4
Fe	1.6	1.2	1.8	1.4
Al	-	6.4	9.5	5.3
Sb	0.3	0.4	0.5	0.4

**Table 3:** Composition of studied locations on MC sculpture (Figure 2) obtained by portable XRF.

Area/Composition wt %	1	2	3
Cu	75.8	76.1	71.1
Pb	1.4	2.3	1.3
Sn	8.7	9.7	10.6
Zn	3.7	4.4	4.3
Fe	2.4	4.0	6.3
Al	7.6	3.1	6.7
Ni	0.1	0.1	0.1

**Table 4:** Raman bands, analyzed on patina at different positions on statue MC (Figure 7) and literature data [36]. Letters next to a number denote strength of the band: vw (very weak), w (weak), s (strong), and vs (very strong).

Point 1 (a)	Point 2 (b)	Point 3 (c)	Langite [36]	Posnjakite [36]
191 (w)		191 (w)	194	195
246 (w)		246 (w)	241	241
386 (w)		386 (w)	391	386
479 (w)		479 (w)	481	482
610 (w)		610 (w)	609	609
974 (s)		974 (s)	974	972
1102 (w)		1102 (w)	1102	1105
1359 (s)	1359 (s)	1359 (s)		
1598 (s)	1598 (s)	1598 (s)		
3402 (w)		3402 (w)	3405	3405
3586 (w)		3586 (w)	3587	3588

**Table 5:** EIS parameters obtained by fitting impedance data in Figure 9.

Sample	*R*_1_/kΩ cm^2^	*Q*_1_/μS s^n^ cm^−2^	*n*_1_	*R*_2_/kΩ cm^2^	*Q*_2_/μS s^n^ cm^−2^	*n*_2_
NI1	6.69	3.31	0.55	146	1.04	0.75
NI2	1.97	2.71	0.84	60.2	3.02	0.91
NI4	1.59	2.83	0.56	105	31.0	0.50

**Table 6:** EIS parameters obtained by fitting impedance data in Figure 11.

Sample	*R*_1_/kΩ cm^2^	*Q*_1_/μS s^n^ cm^−2^	*n*_1_	*R*_2_/kΩ cm^2^	*Q*_2_/μS s^n^ cm^−2^	*n*_2_	*R*_3_/kΩ cm^2^	*Q*_3_/μS s^n^ cm^−2^	*n*_3_
MC2	0.19	12.5	0.51	2.69	98.5	0.76	63.7	98.4	0.42
MC3	0.47	3.25	0.51	1.01	3.69	0.55	76.3	129	0.40

## Data Availability

The data that support the findings of this study are available from the corresponding author upon reasonable request.
